# An Efficient Pulse Circuit Design for Magnetic Stimulation with Diversified Waveforms and Adjustable Parameters

**DOI:** 10.3390/s24123839

**Published:** 2024-06-13

**Authors:** Xiao Fang, Tao Zhang, Yaoyao Luo, Shaolong Wang

**Affiliations:** 1 College of Nuclear Technology and Automation Engineering, Chengdu University of Technology, Chengdu 610059, China; 2 High Field Magnetic Resonance Brain Imaging Laboratory of Sichuan, Chengdu 611731, China; 3 Key Laboratory for NeuroInformation of Ministry of Education, School of Life Science and Technology, University of Electronic Science and Technology of China, Chengdu 611731, China; 4 State Key Laboratory of Advanced Electromagnetic Engineering and Technology, Huazhong University of Science and Technology, Wuhan 430074, China

**Keywords:** pulsed E-field, multi-waveforms, adjustable pulse parameters, circuit design

## Abstract

As a noninvasive neuromodulation technique, transcranial magnetic stimulation (TMS) has important applications both in the exploration of mental disorder causes and the treatment of mental disorders. During the stimulation, the TMS system generates the intracranial time-varying induced E-field (E-field), which alters the membrane potential of neurons and subsequently exerts neural regulatory effects. The temporal waveform of the induced E-fields is directly related to the stimulation effect. To meet the needs of scientific research on diversified stimulation waveforms and flexible adjustable stimulation parameters, a novel efficient pulse magnetic stimulation circuit (the EPMS circuit) design based on asymmetric cascaded multilevel technology is proposed in this paper. Based on the transient analysis of the discharge circuit, this circuit makes it possible to convert the physical quantity (the intracranial induced E-field) that needs to be measured after magnetic stimulation into easily analyzable electrical signals (the discharge voltage at both ends of the stimulation coil in the TMS circuit). This EPMS circuit can not only realize monophasic and biphasic cosine-shaped intracranial induced E-fields, which are widely used in the market, but also realize three types of new intracranial induced E-field stimulation waveform with optional amplitude and adjustable pulse width, including monophasic near-rectangular, biphasic near-rectangular and monophasic/biphasic ladder-shaped stimulation waveform, which breaks through the limitation of the stimulation waveform of traditional TMS systems. Among the new waveforms produced by the EPMS circuit, further research was conducted on the dynamic response characteristics of neurons under the stimulation of the biphasic four-level waveform (the BFL waveform) with controllable parameters. The relationship between TMS circuit parameters (discharge voltage level and duration) and corresponding neural response characteristics (neuron membrane potential change and neuronal polarizability ratio) was explained from a microscopic perspective. Accordingly, the biological physical quantities (neuronal membrane potential) that are difficult to measure can be transformed into easily analyzable electrical signals (the discharge voltage level and duration). Results showed that compared with monophasic and biphasic cosine induced E-fields with the same energy loss, the neuron polarization ratio is decreased by 54.5% and 87.5%, respectively, under the stimulation of BFL waveform, which could effectively enhance the neuromodulation effect and improve the stimulation selectivity.

## 1. Introduction

In recent years, transcranial magnetic stimulation (TMS) has become a research hotspot in the field of biomedical engineering due to its superior penetration characteristics, and it has shown a strong application prospect in the field of mental disorder treatment [1,2,3,4,5,6,7]. During the treatment of TMS, the stimulation coil is placed near the human head, and the time-varying current in the stimulation coil generates a spatial pulse magnetic field. Since the conductivity and permeability of human tissues are not zero, the pulse magnetic field will produce induced E-fields (E-fields) within the brain tissues, which will cause the depolarization of the neurons, affect the metabolic activities of the brain tissue and then play a role in neural regulation. The topology of the TMS circuit determines the temporal distribution of the intracranial induced pulsed E-fields. Through the specific design of the TMS circuit, the temporal distributions of the pulsed E-field can be tracked, and it is possible to control the neuromodulation effect, which has great potential for scientific research.

Many researchers have studied the pulsed E-field generating circuit [8,9,10,11,12,13]. Baker of the University of Sheffield developed the first medical pulsed magnetic field generator, which could induce a cosine pulsed E-field within the human head [14]; Ueno from Kyushu University proposed a biphasic pulsed E-field generator that could generate biphasic cosine pulsed E-field by anti-paralleling diodes at both ends of the discharge switch [15]; Researchers from the Munich University of Technology used an IGBT full-bridge module to realize the stimulus current with flat-top, which enriches the diversity of the pulsed E-field waveform. However, due to the small energy storage capacitance in this circuit, the flat-top fluctuation of the pulse current was large, and the pulse width was difficult to adjust [10]. Researchers from Duke University and Columbia University proposed a parameter-controllable pulsed E-field generation circuit, which can generate an approximately rectangular pulsed E-field in the brain. Fully controlled switching devices were used in this circuit to realize adjustable pulse width of an induced pulsed E-field [8,11,12,13], and it could generate up to four levels of the pulsed E-field within one pulse cycle; Xiong Hui of Tianjin University of Technology proposed an energy-saving pulsed E-field generator which could reduce the power consumption and improve the working efficiency [16]. However, most of the pulsed E-field generating circuits were designed to generate one or two types of pulsed E-field waveform, and the adjustable parameters of the stimulation waveform are limited, which cannot meet the need for diversified stimulation waveforms.

With the gradual deepening of the exploration process of the nervous system, the requirement for the TMS circuit is growing higher. Due to the complex causes of mental disorders, the in-depth mechanism of the induced pulsed E-field’s effect in the treatment of mental diseases is still in the exploration stage. The optimum induced pulsed E-field waveform that brings the best stimulation effect has not been determined [17]. The parameters of the pulsed E-field waveform, including the amplitude, pulse width, polarity, and duration of each phase, affect the neuromodulation effect from different aspects [12,18,19]. Different research purposes have different requirements for the waveform of the pulsed E-field; e.g., in the measurement of nerve conduction and the theoretical modeling of neurons, it needs to induce an approximate rectangular induced E-field in the brain to derive the time-intensity curves of different neurons to obtain the time constant of neuron membrane [13]. Therefore, it is necessary to diversify the pulsed E-field waveform by designing the TMS circuit topology and to create possibilities for scientific research [18,19,20,21,22,23,24,25]. On the other hand, it is valuable to realize flexible pulse parameters so that researchers can dynamically adjust the stimulation parameters according to the feedback of different patients during the stimulation process [26,27,28].

Considering the design requirement of TMS circuits, a novel efficient pulse circuit (the EPMS circuit) design based on asymmetric cascaded multilevel technology is proposed in this paper. First, the principle of the EPMS circuit is analyzed in detail. Then, three kinds of new pulsed E-field waveforms with optional amplitude and adjustable pulse width generated by the EPMS circuit are introduced and discussed. Finally, among the available new pulsed E-field waveform generated by the EPMS circuit, the biphasic four-level pulsed E-field waveform with controlled parameters (the BFL waveform) is taken as an example to further analyze the neuron response characteristics under magnetic stimulation.

## 2. Methods

### 2.1. Transient Analysis of the Discharge Circuit

**Figure 1:**
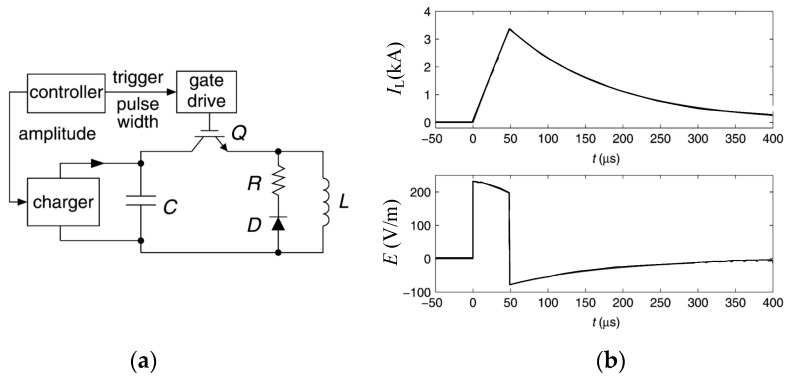
The monophasic pulsed E-field generating circuit: (**a**) Description of the circuit topology; (**b**) The pulsed stimulation current and the intracranial pulsed E-field.

Figure 1a shows the monophasic pulsed E-field generating circuit. In Figure 1a, the energy storage capacitance is *C*; the inductance of the stimulation coil is *L*; *r* is the equivalent resistance of the stimulation coil and the discharge switch. The *R* and *D* represent the freewheeling resistance and the freewheeling diode, respectively. Figure 1b shows the pulsed stimulation current and the intracranial pulsed E-field generated by the monophasic pulsed E-field generating circuit.

Because the conductivity and permeability of human biological tissues are rather small, the induced pulsed E-field decays rapidly within the brain. The targeted area corresponding to mental diseases is generally non-superficial and is located at a depth of 20 mm or deeper under the skull. If the ideal treatment effect is to be achieved, the induced pulsed E-field at the targeted area must have a certain intensity. Therefore, the pulsed current used in biomedical therapy usually has the characteristics of high amplitude (>2.5 kA) and narrow pulse width. At the first rising edge of the pulsed current (in the first quarter of the resonance period), the pulsed current increases approximately linearly, as shown in Figure 1b.

According to the discharge transient analysis of the monophasic pulsed E-field generating circuit in Figure 1a, the temporal expressions of the pulsed current *I_Lcoil_*(*t*) and the induced pulsed E-field *E*(*t*) can be obtained, as shown in Equations (1) and (2).

(1) $$I_{L_{\mathrm{coil}}}(t)=\left\{\begin{array}{l} \frac{V_{C}}{\omega L}\sin(\omega t)\times e^{\left(-\sigma t\right)},(0<t\leq t_{p})\\ \frac{V_{C}}{\omega L}\sin(\omega t_{p})\times e^{\left(-\frac{\left(t-t_{p})\times (r+R\right)}{L}-\sigma t_{p}\right)},(t>t_{p}) \end{array}\right.$$

(2) $$E(t)=\left\{\begin{array}{l} \delta \frac{V_{C}}{\omega L}\left[\cos(\omega t)-\frac{\sigma }{\omega }\sin(\omega t)\right]\times e^{\left(-\sigma t\right)},(0<t\leq t_{p})\\ -\delta \frac{V_{C}(R+r)}{\omega L^{2}}\sin(\omega t_{p})\times e^{\left(-\frac{\left(t-t_{p})\times (r+R\right)}{L}-\sigma t_{p}\right)},(t>t_{p}) \end{array}\right.$$

(3) $$\omega =\sqrt{\frac{1}{LC}-{\left(\frac{r}{2L}\right)}^{2}},\sigma =\frac{r}{2L}$$

where *V*_C_ represents the voltage on the energy storage capacitor; *R* is the freewheeling resistance, *L* is the inductance of the stimulation coil, and *R* is the equivalent series resistance of the circuit; *T*_p_ is equal to *T*/4, which is related to the resonant period *T* of the discharge circuit, *T* = *2π√LC*; *δ* represents the proportional coupling coefficient and is affected by the geometry of the stimulation coil, the position of the stimulation coil relative to the human head, the biological structure of target tissues and other relative factors. In this paper, the value of *δ* is 3.2 × 10^−6^ (V/m)/(A/S) [8]. *ω* represents the resonant frequency, *σ* represents an intermediate variable.

When the equivalent series resistance *R* is small, and the energy storage capacitance *C* is large, Equation (2) can be simplified to Equation (4).

(4) $$E(t)=\left\{\begin{array}{l} \delta \frac{V_{C}}{L},(0<t\leq t_{p})\\ -\delta \frac{V_{C}R\cdot t_{p}}{L^{2}}\times e^{-\frac{(t-t_{p})\times R}{L}},(t>t_{p}) \end{array}\right.$$

A key conclusion can be drawn from the simplified Equation (4): when *t* < *Tp*, the voltage level *V_C_* of the energy storage capacitor discharging the stimulation coil determines the amplitude of the intracranial induced pulsed E-field, and the turn-on time of the discharge switch is the pulse width (PW) of the induced pulsed E-field. This conclusion shows that we can analyze the induced pulsed E-field by measuring the discharge voltage at both ends of the stimulation coil in the TMS circuit.

### 2.2. Working Principles of the EPMS Circuit

#### 2.2.1. Topology of the EPMS Circuit

**Figure 2:**
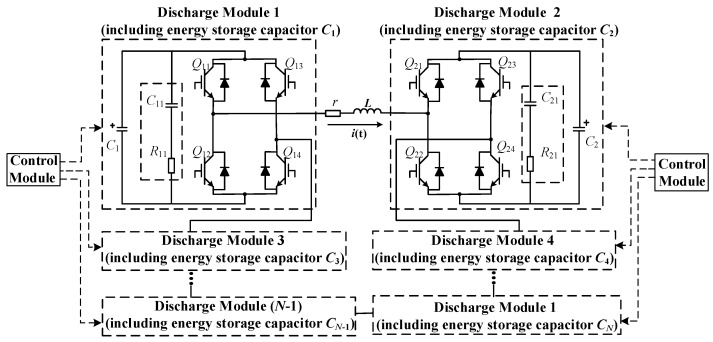
The topology of the EPMS circuit.

Based on the analysis of the monophasic pulsed E-field generating circuit, we propose the EPMS circuit. The topology of the EPMS circuit is shown in Figure 2. The EPMS circuit is composed of a magnetic stimulation coil and *N*-independent discharge modules. Each independent discharge module includes an energy storage capacitor and an IGBT full-bridge structure. The *RC* buffer circuits (*C*_11_, *C*_21_*,* and *R*_11_~*R*_12_ in Figure 2) are paralleled at both ends of the full-bridge structure of each discharge module to absorb part of the current flowing through IGBT when the discharge switch is disconnected, and it can also suppress the collector-emitter voltage spike of IGBT caused by parasitic inductance [11,12,13,29,30,31,32]. The solid line with the arrow indicates the positive direction of the pulsed current *i*(t).

#### 2.2.2. Different Working Modes of the EPMS Circuit

Before the stimulation, each energy storage capacitor in the EPMS circuit has been charged to the preset voltage value by its power supply module. At the beginning of stimulation therapy, the control module provides the driving signal to the IGBT and the energy storage capacitors in each module are cascaded through the IGBT full bridge to discharge the stimulation coil and generate a pulsed current in the coil.

The turn-on state of the IGBT switch in the *N*-independent discharge modules of the EPMS circuit determines the combination state of the discharge modules and then determines the discharge voltage level of the energy storage capacitor that discharges the coil.

According to the transient analysis of the monophasic pulsed E-field generating circuit in Section 2.1, the discharge voltage level of the energy storage capacitor is proportional to the intracranial induced pulsed E-field level. The more discharge modules in the EPMS circuit, the more combinations of energy storage capacitors are available, and the more intracranial induced pulsed E-field levels that can be formed.

**Figure 3:**
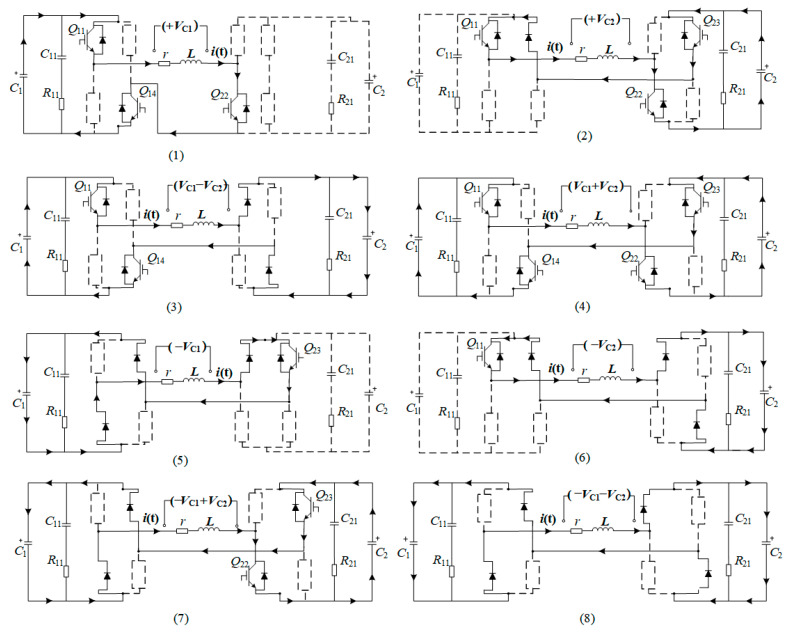
Eight kinds of non-zero discharge voltage generated by the EPMS circuit at *N* = 2. The black arrows indicate the current direction.

Take the EPMS circuit when *N* = 2 as an example: set the two energy storage capacitors in the discharge module as *C*_1_ and *C*_2_, respectively. The preset voltages on the energy storage capacitor are *V_C_*_1_ and *V_C_*_2_ (*V_C_*_1_ > *V_C_*_2_). *L* is the equivalent inductance of the stimulation coil. *R* is the equivalent resistance in the pulsed discharge circuit. The available discharge voltages under different turn-on states of the IBGT in the two full-bridge structures are shown in Figure 3.

Figure 3 shows the different combinations of energy storage capacitors achieving eight non-zero discharge voltage levels. Assuming the positive direction of voltage on the coil is shown by the arrow in Figure 2, there are nine voltage levels available in the EPMS circuit at *N* = 2: +*V*_C1_, +*V*_C2_, *V*_C1_ − *V*_C2_, (*V*_C1_ + *V*_C2_), −*V*_C1_, −*V*_C2_, *V*_C2_ − *V*_C1_, (−*V*_C1_ − *V*_C2_), and zero voltage. When the EPMS circuit is switched to each combination mode in Figure 3, it is equivalent to an independent monophasic discharge circuit discussed in Section 2.1. If the discharging duration of each combination mode is far less than 1/4 of its resonance period, the voltage level of the induced E-field and discharge circuit can be satisfied: *E* = *δ·Vc*/*L*.

**Figure 4:**
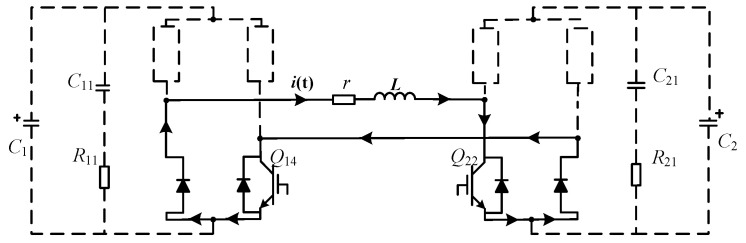
The zero discharge voltage at *N* = 2. The black arrows indicate the current direction.

When the pulsed current flows through the IGBT or diode at the same position of the left and right bridge arms of the full-bridge module, the energy storage capacitor in the module is bypassed and does not participate in the discharge. If both capacitors are bypassed, the induced pulsed E-field of zero-level is achieved, as shown in Figure 4.

On the other hand, the current in an inductance cannot leap. The slope of the pulse current is proportional to the voltage on the coil. When the energy storage capacitor discharges the coil, the equation *V_C_* = *V_L_* = *L*·d*i*/d*t* is satisfied, so there is *E* = *δ*·*V*c/*L* = *δ*·d*i*/d*t,* which means the slope of pulsed current d*i*/d*t* is proportional to the level of intracranial induced pulsed E-field and the proportional coefficient is *δ*.

When *N* = 2, the discharging voltage level corresponding to the rising edge of the current can be selected from {+*V*_C1_, +*V*_C2_, (*V*_C1_ + *V*_C2_), (*V*_C1_ −*V*_C2_)} and the discharge voltage level corresponding to the falling edge can be selected from {−*V*_C1_, −*V*_C2_, −(*V*_C1_ + *V*_C2_), (*V*_C2_ − *V*_C1_)}.

**Figure 5:**
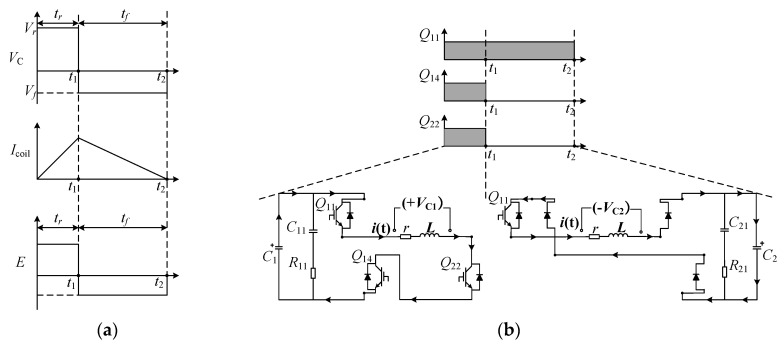
The discharge voltage levels corresponding to the rising and falling edges of the pulsed current are *V_r_* and *V_f_*, respectively. The duration of the rising and falling edge of the pulsed current are *t_r_* and *t_f_*, respectively. (**a**) The corresponding relationships between the discharge voltage *V*_C_ on the energy storage capacitor, the stimulation current *I_coil_* and the pulsed E-field *E*; (**b**) The control sequence of the IGBTs when *V_r_* = +*V_C_*_1_ and *V_f_* = −*V_C_*_2_.

Take generating a monophasic pulsed current waveform as an example. The corresponding relationships between the discharge voltage on the energy storage capacitor, the stimulation current, and the pulsed E-field are shown in Figure 5a. The discharge voltage levels corresponding to the rising and falling edges of the pulsed current are expressed as *V_r_* and *V_f_*, respectively. The duration of the rising and falling edge of the pulsed current is expressed as *t_r_* and *t_f_*, respectively. Assuming the *V_r_* = +*V*_C1_ and *V_f_* = −*V*_C2_, the control sequence of the IGBT switch is shown in Figure 5b.

Since the discharge switches used in the EPMS circuit are full-controllable IGBT, the pulse width of the induced pulsed E-field waveform generated by the EPMS circuit can be adjusted flexibly. The maximum duration of each level of the induced pulsed E-field is limited by the resonant period. To increase the adjustable range of the pulse width and reduce the requirement of IGBT switching frequency, the energy storage capacity *C* used in the EPMS circuit is larger than that of a traditional TMS circuit. The driving signal of IGBT is obtained by the PWM method in the control module to accurately control the on-off state of each switch and obtain the required waveform.

### 2.3. Implementation Scheme of the EPMS Circuit

The waveform of the intracranial induced pulsed E-field generated by the EPMS circuit is not only related to the circuit topology and the turn-on state of the discharge switch but is also affected by the components used in the circuit. Taking the module number of *N* = 2 as an example, based on our previous experience of designing pulsed electromagnetic field generators in the Wuhan National High Magnetic Field Center (WHMFC), we designed a modularized charging circuit for the energy storage capacitors of the EPMS circuit and then proposed a component selection scheme for the EPMS circuit which is in line with the engineering practice and can prepare for the next stage of prototype development and biological experiment.

#### 2.3.1. Charging Method for the EPMS Circuit

**Figure 6:**
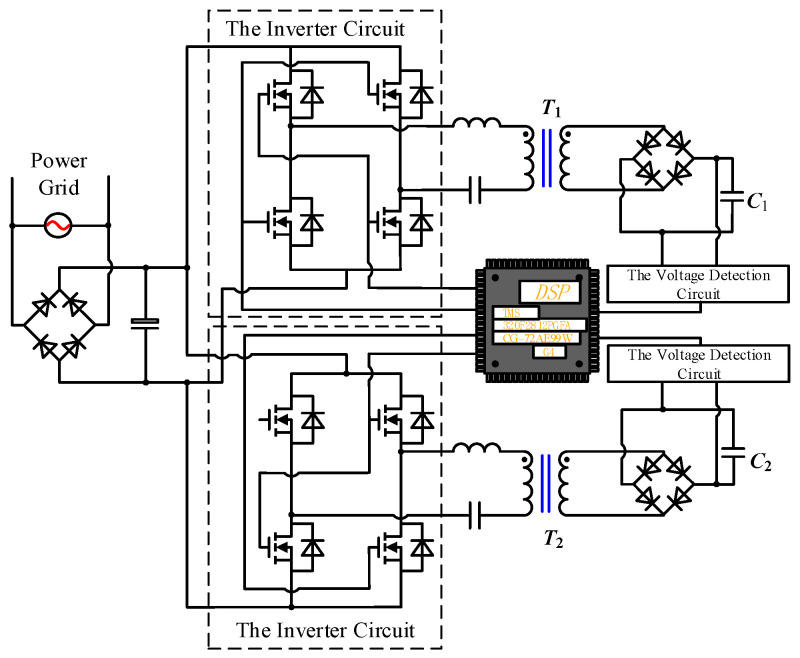
The modularized charging circuit for *C*_1_ and *C*_2_.

The constant current charging method has the advantages of high efficiency and causing a slighter impact on the power grid. The high-frequency series resonant constant current charging method can be adapted to charge the energy storage capacitor. The main circuit diagram of the charging part is shown in Figure 6. The power frequency alternating current changes to the high-frequency alternating current through the rectification circuit and inversion circuit. Then, the boost transformer T1 and T2, as well as the rectification circuit, generate direct voltage with high amplitude to charge the energy storage capacitor. Since the designed EPMS circuit contains two independent energy storage capacitors, *C*_1_ and *C*_2_, the power frequency alternating current runs into two relatively independent charging sets after the rectification circuit to supply power for *C*_1_ and *C*_2_, respectively. The signal processor is implemented by DSP (320F2812FGFA, Texas Instruments, Dallas, TX, USA) to generate the gate control signals of each switch device. The rated voltage and capacity of the two boost transformers are 4200 V and 10.5 kVA, the ratio between the primary and secondary winding turns is 1:14, and the leakage inductance is 24 μH. To reduce the system volume, the transformer’s leakage inductance is used in the series resonant circuit to substitute for the physical inductance. The core part of the sampling module is an 18-bit chip (AD7609, ADI Company, Melville, NY, USA), and the sampling frequency is 5 kHz.

#### 2.3.2. Components Selection of the EPMS Circuit

As we can see from Figure 2, the EPMS circuit mainly includes three parts: the energy storage capacitor, the IGBT switch and its buffer circuit, and the stimulation coil.

(1) Energy storage capacitor: C_1_ = C_2_ = 370 μF (# (2) 39738, General Atomics). To increase the pulse width adjustment range, the capacitance value is larger than the capacitance value used in the traditional pulsed electric field generating circuit. Before the discharge process begins, the voltage on the energy storage capacitors C_1_ and C_2_ are charged to *V*_C1_ = 1800 V and *V*_C2_ = 1200 V, respectively.

(2) IGBT switch and its buffer circuit: IGBT module 5SNA0800J450300 (ABB) is an ideal choice for the discharge switch in the EPMS circuit. Its collector-emitter rated voltage is 4500 V, and the rated DC is 800 A. Normally, the pulsed current amplitude in biomedical research is about 2 kA, which is higher than the current rating of the IGBT module. However, when the IGBT module has a very short turn-on time and is equipped with a parallel buffer circuit, the IGBT module in the pulsed discharge circuit can transiently withstand 10 times its rated current in a brief time [11,12,13,29,30,31]. Each IGBT full-bridge modular in the EPMS circuit has an RC buffer circuit parallel to it (as shown in Figure 2) to absorb the current during the shutdown process and suppress the IGBT voltage spike caused by parasitic inductance. This method has been used many times in the design of biomedical discharge circuits [11,12,13,29,30,31,32]. To effectively limit the collector-emitter voltage and minimize the power loss on R11 and R21, the buffer parameter is set as C_11_ = C_12_ = 5 μF(2 × 2.5 μF. Polypropylene film capacitor, C4DRYAQ4250AA0J, KEMET), R_11_ = R_12_ = 0.05 Ω (0505MR050LWWS, Vishay/Thin film resistor). Due to the existence of the buffer circuit, even if C_1_ and C_2_ are charged to their maximum voltage (*V*_C1_ = *V*_C2_ = 2300 V), the voltage spike generated when IGBT is turned off can still be limited below its rated voltage to ensure the safe operation of IGBT.

(3) Stimulation coil: the design of the EPMS circuit has no special restrictions on the stimulation coil. In this paper, the equivalent inductance and resistance of the stimulation coil are set at 30 μH and 16 mΩ based on our previous coil design results. The stray resistance of the circuit is assumed to be 9 mΩ. In Figure 2, L = 30 μH, R = 16 m Ω + 9 m Ω = 25 m Ω.

## 3. Results

In biomedical pulsed E-field generators, the pulsed current is usually divided into monophasic pulsed current and biphasic pulsed current based on whether the current has both positive and negative phases within one pulse period. Similarly, the intracranial induced pulsed E-field can be divided into monophasic and biphasic waveforms. Due to the existence of the full-bridge module, the EPMS circuit can not only generate monophasic and biphasic cosine-shaped intracranial induced pulsed E-fields, which are commonly used presently, but can also generate a variety of new intracranial induced pulsed E-field waveforms.

For the convenience of analysis and comparison, the new intracranial induced pulsed E-field waveforms generated by the EPMS circuit are divided into three types: Type 1: the monophasic approximate rectangular pulsed E-field waveform; Type 2: the biphasic approximate rectangular pulsed E-field waveform and Type 3: the ladder-shaped pulsed E-field waveform. The simulations are carried out on the MATLAB2021a platform.

### 3.1. Three Types of New E-Field Waveform Generated by the EPMS Circuit

#### 3.1.1. Type 1: The Monophasic Approximate Rectangular Pulsed E-Field

When the waveform of the pulsed current is a monophasic approximate triangle wave, the rising time of the current is *t_r_*, the falling time of the current is *t_f_*, and the corresponding discharge voltage levels of the rising and falling edge are *V_r_* and *V_f_*, respectively. Since the pulsed current must be zero at the end of the pulse, the constraint condition can be described as Equation (5):

(5) $$V_{r}\times t_{r}+V_{f}\times t_{f}=0$$

where *V_r_* ⸦ {+*V*_C1_, +*V*_C2_, (+*V*_C1_+*V*_C2_), (+*V*_C1_ − *V*_C2_)}, *V_f_* ⸦ {−*V*_C1_, −*V*_C2_, −(*V*_C1_ + *V*_C2_), (*V*_C2_ − *V*_C1_)}.

**Figure 7:**
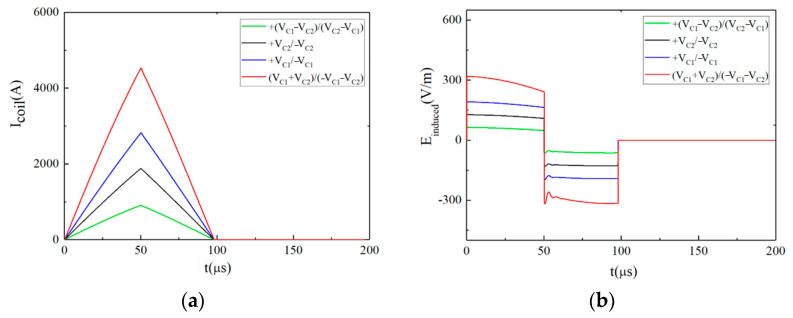
When *t_r_* = *t_f_* = 50 μs, the pulsed waveforms generated by the EPMS circuit with different *V_r_* and *V_f_*: (**a**) The monophasic near-triangular pulsed current; (**b**) The monophasic intracranial induced E-field with adjustable amplitude.

**Figure 8:**
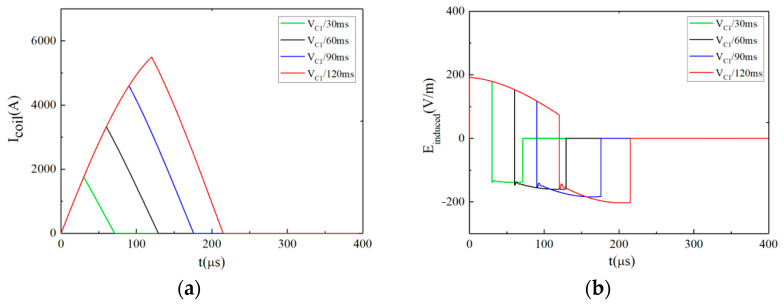
When *V_r_* = *V_C_*_1_ = 1800 V, *V_f_* = −*V_C_*_2_ = −1200 V, the pulsed waveforms generated by the EPMS circuit with different *t_r_* and *t_f_*: (**a**) The monophasic near-triangular pulsed current; (**b**) The monophasic intracranial induced E-field with adjustable amplitude.

When the pulsed current is a monophasic approximate triangular wave, the temporal distribution of the intracranial induced pulsed E-field is approximately rectangular. The corresponding relationships between the discharge voltage on the energy storage capacitor, the stimulation current and the pulsed E-field are shown in Figure 7 and Figure 8.

Figure 7a,b show the pulsed current and its induced pulsed E-field under different discharge voltage levels (the pulse width is fixed at *t_r_* = *t_f_* = 50 μs), respectively. The voltage levels of *Vr* and *Vf* in each waveform are shown in the upper right corner of Figure 7. Figure 7b shows that the amplitude of the intracranial induced pulsed E-field is adjustable. According to Equation (5), when *N* = 2, the EPMS circuit can generate 4 × 4 kinds of monophasic approximately rectangular E-field waveforms with different amplitudes.

Figure 8a,b show the pulsed current and its induced pulsed E-field when *V_r_* = *V_C_*_1_ = 1800 V, *V_f_* = −*V_C_*_2_ = −1200 V and *t_r_*⸦ [30 μs, 60 μs, 90 μs, 120 μs]. The *t_r_* is shown in the upper right corner of Figure 8. Figure 8b shows the adjustable pulse width of the intracranial induced pulsed E-field. When *V_r_*, *V_f_*, and *tr* are determined, *t_f_* can be obtained according to Equation (5).

#### 3.1.2. Type 2: The Biphasic Approximately Rectangular Pulsed E-Field

When the pulsed current waveform is biphasic approximate triangular, there are two cases: (1) if the slope of the current before and after the zero-crossing point is not changed, one pulsed current period can be divided into three segments, namely one rising edge and two falling edges. (2) When the slope of the current changes before and after the zero-crossing point, one pulsed current period can be divided into four segments, namely two current rising edges and two current falling edges. In the EPMS circuit, when *N* = 2, it can provide four positive and four negative discharge voltage levels (excluding zero voltage), so it can not only realize the first kind of biphasic approximate triangular wave pulsed current but also produce the second kind of biphasic approximate triangular pulsed current. The second kind of biphasic approximate triangular pulsed current and its induced pulsed E-fields will be introduced in detail in the next section. Here, we mainly introduce the first kind of biphasic approximate triangular pulsed current and its induced pulsed E-fields. As discussed in the previous section, the EPMS circuit can produce four positive and four negative induced E-field levels in one current pulse cycle, corresponding to four positive and four negative slopes of the pulsed current.

On this basis, the pulsed current within one cycle can be divided into four parts, including two rising edges and two falling edges, so that a biphasic four-level pulsed E-filed waveform with controlled parameters (the BFL waveform) can be induced in the brain. The pulsed current that produces the BFL waveform is called biphasic four-segment current, which belongs to the second kind of biphasic approximate triangular pulsed current mentioned in Section 3.1.2.

There are two stimulus modes in biomedical research: anterior to post current flow (AP) and post to prior current flow (PA). In AP and PA modes, the peak current of the first phase is negative and positive, respectively [10]. Both AP and PA biphasic stimulation modes can be realized in the EPMS circuit. In this paper, the AP mode is taken as an example to analyze the stimulation effect of biphasic pulsed current, which means the pulsed current first increases in the opposite direction to the negative peak of the first phase and then increases in the positive direction to the positive peak of the second phase.

Suppose the duration of the rising segment of biphasic pulsed current is *t_r_*, and the duration of the first and second falling edges are *t_f_*_1_ and *t_f_*_2_, respectively. The corresponding discharge voltage levels of the rising edge and the falling edge are *V_r_*, *V_f_*_1_, and *V_f_*_2_, respectively. The constraint within one pulse cycle is shown in Equation (6).

(6) $$V_{r}\times t_{r}+(V_{f1}\times t_{f1}+V_{f2}\times t_{f2})=0$$

where *V_r_* {+*V*_C1_, +*V*_C2_, (*V*_C1_ + *V*_C2_), (*V*_C1_ − *V*_C2_)}; *V_f_*_1_, *V_f_*_2_ ⸦{−*V*_C1_, −*V*_C2_, −(*V*_C1_ + *V*_C2_), (*V*_C2_ − *V*_C1_)}.

**Figure 9:**
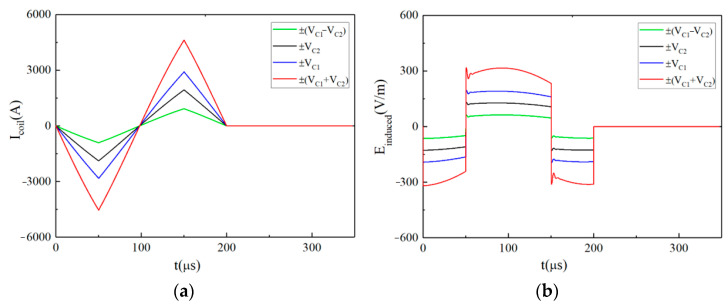
When *t_r_* = 100 μs, *t_f_*_1_ = *t_f_*_2_ = 50 μs, the pulsed waveforms generated by the EPMS circuit with different *V_r_*, *V_f_*_1_, and *V_f_*_2_: (**a**) The biphasic near-triangular pulsed current; (**b**) The biphasic intracranial induced E-field with adjustable amplitude.

Figure 9a,b show the pulsed current and its induced pulsed E-field under different discharge voltage levels (the pulse width is fixed at *t_r_* = 100 μs, *t_f_*_1_ = *t_f_*_2_ = 50 μs). The corresponding voltage levels of *V_r_*, *V_f_*_1_, and *V_f_*_2_ in each waveform are shown in the upper right corner. Figure 9b shows that the amplitude of the intracranial induced pulsed E-field is adjustable. According to Equation (6), the EPMS circuit can generate 4 × 4 kinds of biphasic approximately rectangular E-field waveforms with different amplitudes.

**Figure 10:**
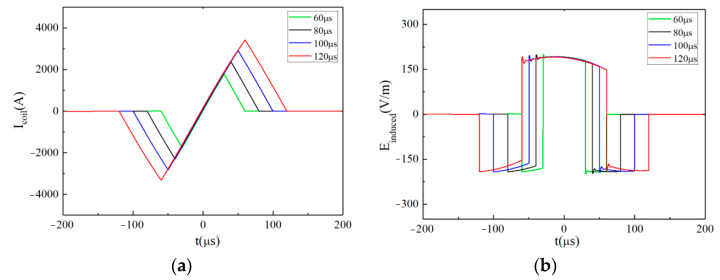
When *V_r_* = *V*_C1_ = 1800V, *V_f_*_1_ = *V_f_*_2_ = −*V*_C1_ = −1800 V, the pulsed waveforms generated by the EPMS circuit with different *t_r_*, *t_f_*_1_, and *t_f_*_2_: (**a**) The biphasic near-triangular pulsed current; (**b**) The biphasic intracranial induced E-field with adjustable amplitude.

Figure 10a,b show the pulsed current and its induced pulsed E-field when *V_r_* = *V*_C1_ = 1800 V, *V_f_*_1_ = *V_f_*_2_ = −*V*_C1_ = −1800 V and *t_r_* ⸦ [60 μs, 80 μs, 100 μs, 120 μs]. The *t_r_* is shown in the upper right corner of Figure 10. Figure 10b shows the adjustable pulse width of the intracranial induced pulsed E-field. When *V_r_*, *V_f_*_1_, *V_f_*_2_, and *t_r_* are determined, *t_f_*_1_ and *t_f_*_2_ can be obtained according to Equation (6).

#### 3.1.3. Type 3: The Ladder-Shaped Pulsed E-Field

Suppose that the rising edge of monophasic and biphasic pulsed current corresponds to the discharge voltage levels of *V_r_*_1_, *V_r_*_2_, *V_r_*_3_, …, *V_r_*_p_ and the falling edge corresponds to the discharge voltage levels of *V_f_*_1_, *V_f_*_2_, *V_f_*_2_, …, *V_f_*_q_. The rising edge of each segment lasts for *t_r_*_1_, *t_r_*_2_, *t_r_*_3_, …, *t_r_*_p_, and the falling edge of each segment lasts for *t_f_*_1_, *t_f_*_2_, *t_f_*_2_, …, *t_f_*_q_:

(7) $$\sum_{i=1}^{i=p}V_{ri}\times t_{ri}+\sum_{i=1}^{i=p}V_{fi}\times t_{fi}=0$$

among them: *V_r_*_1_, *V_r_*_2_, *V_r_*_3_, …, *V_r_*_p_ ⸦ {+*V_C_*_1_, +*V_C_*_2_, (*V_C_*_1_ + *V_C_*_2_), (*V_C_*_1_ − *V_C_*_2_)}, *V_f_*_1_, *V_f_*_2_, *V_f_*_2_, …, *V_f_*_q_ ⸦ {−*V_C_*_1_, −*V_C_*_2_, −(*V_C_*_1_ + *V_C_*_2_), (*V_C_*_2_− *V_C_*_1_)}.

**Figure 11:**
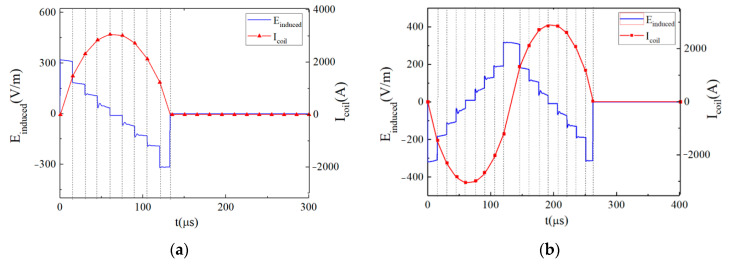
When *N* = 2, the pulsed waveforms generated by the EPMS circuit: (**a**) The monophasic near-triangular pulsed current and its ladder-shaped pulsed E-field waveforms; (**b**) The biphasic monophasic near-triangular pulsed current and its ladder-shaped pulsed E-field waveforms.

In the EPMS circuit, when *N* = 2, the p*_max_* = 4 and q*_max_* = 4. If the current in each pulse cycle was segmented most simply, the monophasic and biphasic approximate triangular stimulus current waveforms can be obtained, which is a special form of the constraint of Equation (8). If the available discharge voltage levels were all included in one pulse cycle, i.e., p = 4 and q = 4, the pulsed current can be further “refined”, and the multilevel induced E-field can be obtained in one pulse cycle, as shown in Figure 11.

As is shown in Figure 11, when two IGBT full-bridge modules are adopted, the EPMS circuit can generate up to nine different levels of pulsed E-field within one pulse period. The pulsed current is approximately a sine wave, while the E-field is a ladder-shaped wave. Since the duration of the E-field is very short for each level, the “segmentation” of the pulsed current is very small when the number of induced E-field grades increases. When the pulsed current is subdivided into several small segments, the whole current waveform will be nearly sinusoidal, and the number of induced E-field levels will be higher (as shown in Figure 11). When the segmentation of the pulsed current is rough, the whole current will be closer to the triangular wave, and the number of induced E-field levels is limited (as shown in Figure 7, Figure 8, Figure 9 and Figure 10).

### 3.2. Further Analysis: A Special Waveform Generated by the EPMS Circuit—The BFL Waveform

#### 3.2.1. Characteristics of the BFL Waveform

As discussed in the previous section, the EPMS circuit can produce four positive and four negative induced E-field levels in one current pulse cycle, corresponding to four positive and four negative slopes of the pulsed current. On this basis, the pulsed current within one cycle can be divided into four parts, including two rising edges and two falling edges, so that a biphasic four-level pulsed E-filed with controlled parameters (the BFL waveform) can be induced in the brain. The pulsed current that produces the BFL waveform is called biphasic four-segment current, which belongs to the second kind of biphasic approximate triangular pulsed current mentioned in Section 3.1.2.

Assume that the rising edges of biphasic four-segment current are *t_r_*_1_, *t_r_*_2_, and the falling edge are *t_f_*_1_, *t_f_*_2_. The corresponding discharge voltage levels of the rising edge and falling edge are *V_r_*_1_, *V_r_*_2_, *V_f_*_1_, and *V_f_*_2_, respectively. The constraint in one pulse period can be expressed as:

(8) $${(V}_{r1}\times t_{r1}+V_{r2}\times t_{r2})+(V_{f1}\times t_{f1}+V_{f2}\times t_{f2})=0$$

where *V_r_*_1_, *V_r_*_2_ ⸦ {+*V_C_*_1_, +*V_C_*_2_, (*V_C_*_1_ + *V_C_*_2_), (*V_C_*_1_ − *V_C_*_2_)}; *V_f_*_1_, *V_f_*_2_ ⸦ {−*V_C_*_1_, −*V_C_*_2_, −(*V_C_*_1_ + *V_C_*_2_), (*V_C_*_2_ − *V_C_*_1_)}.

**Figure 12:**
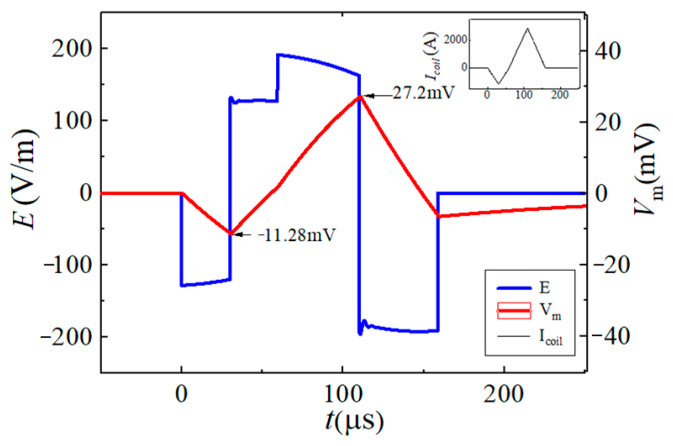
The BFL_A_ waveform when the discharge parameters are −*V_C_*_2_/30 μs, +*V_C_*_2_/30 μs, +*V_C_*_1_/50 μs, −*V_C_*_1_/50 μs.

**Figure 13:**
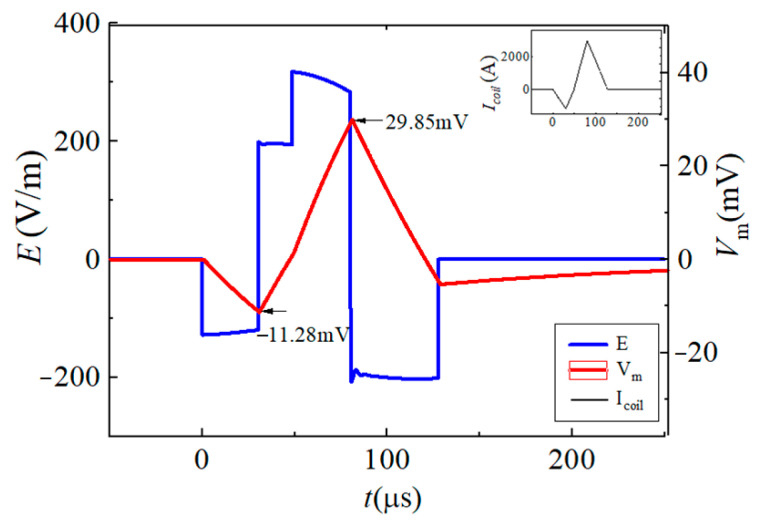
The BFL_B_ waveform when the discharge parameters are −*V*_C2_/30 μs, +*V_C_*_1_/20 μs, (+*V_C_*_1_ + *V*_C2_)/30 μs, −*V_C_*_1_/50 μs.

**Figure 14:**
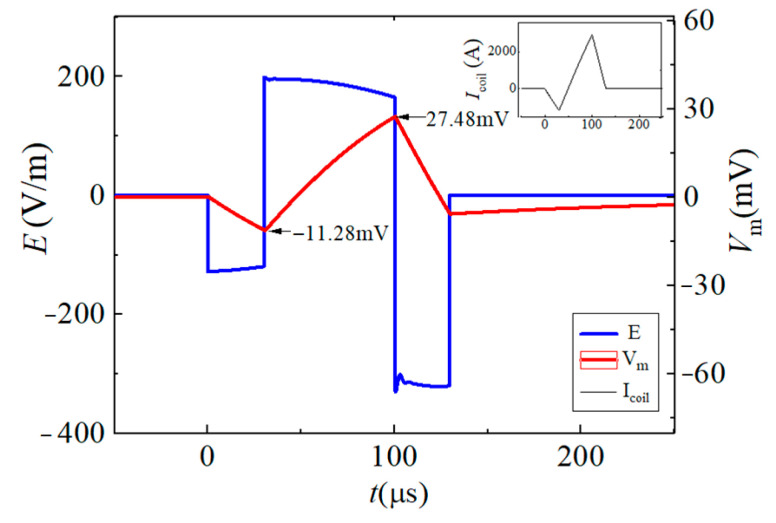
The BFL_C_ waveform when the discharge parameters are −*V_C_*_2_/30 μs, +*V_C_*_1_/20 μs, +*V_C_*_1_/50 μs, −(*V_C_*_1_ + *V*_C2_)/30 μs.

**Figure 15:**
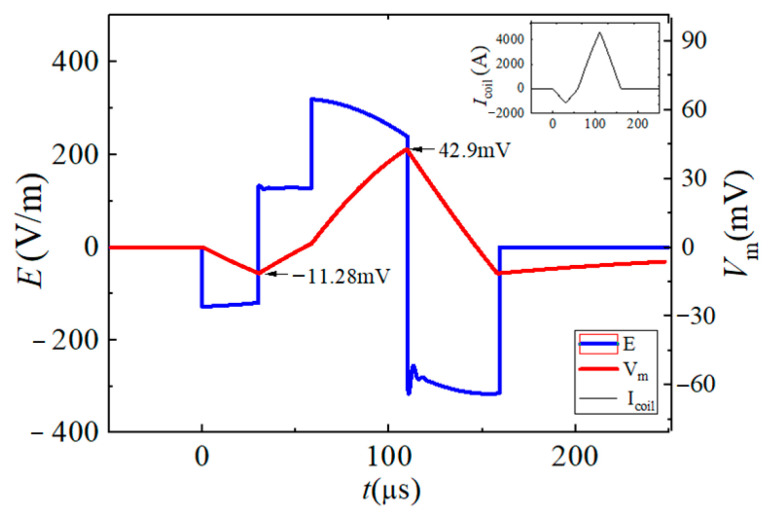
The BFL_D_ waveform when the discharge parameters are −*V*_C2_/30 μs, +*V*_C2_/30 μs, (*V_C_*_1_ + *V*_C2_)/50 μs, (−*V_C_*_1_ − *V*_C2_)/50 μs.

**Figure 16:**
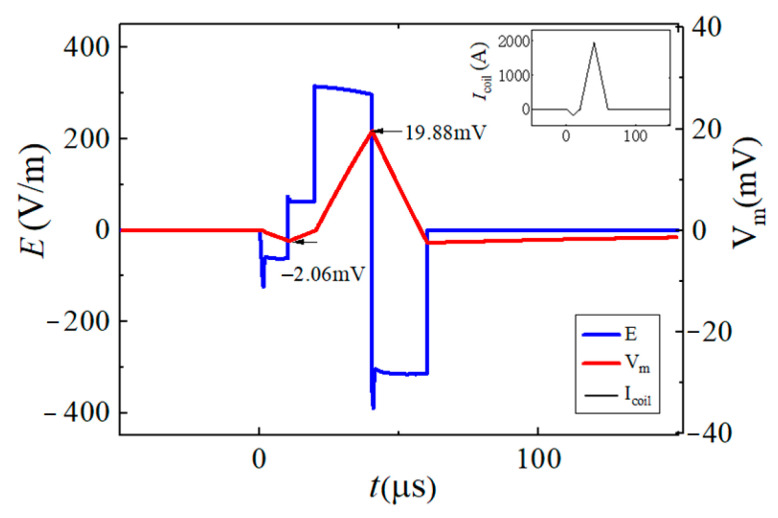
The BFL_E_ waveform when the discharge parameters are −(*V*_C1_ − *V*_C2_)/10 μs, (*V*_C1_ − *V*_C2_)/10 μs, (*V*_C1_ + *V*_C2_)/20 μs, −(*V*_C1_ + *V*_C2_)/20 μs.

**Figure 17:**
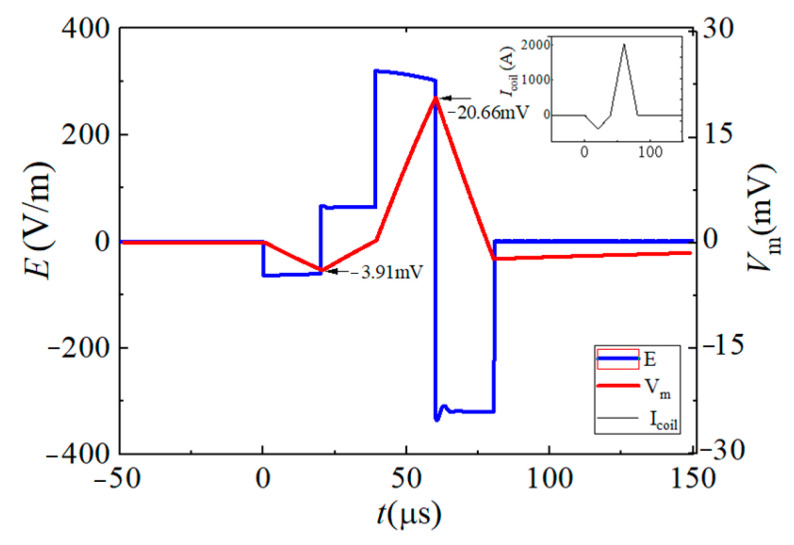
The BFL_F_ waveform when the discharge parameters are −(*V*_C1_ − *V*_C2_)/20 μs, (*V*_C1_ − *V*_C2_)/20 μs, (*V*_C1_ + *V*_C2_)/20 μs, −(*V*_C1_ + *V*_C2_)/20 μs.

In this paper, the neuromodulation effect is assumed to be achieved when the maximum change in neuron membrane potential exceeds 15 mV. By changing the conduction state and turn-on time of the IGBT switch in the EPMS circuit, six representative and effective BFL waveforms are obtained as BFL_A_~BFL_F_. The discharge parameters of each BFL waveform are labeled as *V_f_*_1_/*t_f_*_1_, *V_r_*_1_/*t_r_*_1_, and *V_r_*_2_/*t_r_*_2_, as shown in Figure 12, Figure 13, Figure 14, Figure 15, Figure 16 and Figure 17 and Table 1. *V_f_*_1_, *V_r_*_1_, *V_r_*_2_, and *V_f_*_2_ indicate the discharge voltage level corresponding to the first falling edge, the first rising edge, the second rising edge, and the second falling edge of the pulsed current generating the BFL waveform, respectively. *t_f_*_1_, *t_r_*_1_, *t_r_*_2_, and *t_f_*_2_ indicate the duration of each discharge voltage level.

To reflect the dynamic response of neurons under different pulsed E-fields from a biological view, the Leak Integrate-and-Fire model (the LIF model) is used as the equivalent neuro model to calculate the neuron dynamic responses at the membrane constant of τ_m_ = 150 μs [33,34,35,36]. The neuron response under the stimulation of each BFL waveform is shown in Figure 12, Figure 13, Figure 14, Figure 15, Figure 16 and Figure 17. The blue solid line represents the BFL waveform, the red solid line represents the corresponding neuron membrane potential waveform, and the black solid line in the top right corner represents the corresponding stimulation pulsed current.

#### 3.2.2. Neuron Dynamic Responses under the Stimulation of the BFL Waveform

As measurable parameters in bioelectric experiments, the neuronal polarizability ratio ξ_P_ and the neuron membrane potential change Δ*V*_m_ are important analysis indexes of dynamic neuron responses in biomedical research. The neuronal polarizability ratio ξ_P_ is related to the selectivity of electric stimulation. The change in neuron membrane potential Δ*V*_m_ can characterize the intensity of neural regulation [11,12,13]. Neuronal polarizability ratio ξ_P_ relates to the effect of neuronal hyperpolarization and depolarization, which can be expressed by the ratio of negative to positive peak values of neuronal membrane potential [12].

The neuronal polarizability ratio ξ_P_ and the membrane potential change Δ*V*_m_ are affected by the parameters of an induced pulsed E-field. Since the bioelectricity experiment is difficult to operate, if we can establish the relationships between discharge circuit parameters and neuron response characteristics, the biological physical quantities (neuronal membrane potential) that are difficult to measure can be transformed into easily analyzable electrical signals. In this paper, the BFL waveform is taken as an example to analyze the relationships between discharge circuit parameters and neuron response characteristics.

(1) Neuron Membrane Potential Change Δ*V_m_*

The peak value of neuron membrane potential change under the stimulation of BFL waveform is positively correlated with the maximum value of the product of the discharge voltage level and its duration:

(9) $${\left({∆V}_{m}\right)}_{max}\propto \;{\left((\Pi_{1}^{i=2}V_{ri}\times t_{ri})\cup +(\Pi_{1}^{i=2}\left|\left.V_{fi}\right|\right.\times t_{fi})\right)}_{\max}$$

For BFL_A_~BFL_F_, the combinations of the maximum product of the discharge voltage level and its duration are as follows: +*V*_C1_/50 μs, (*V*_C1_ + *V*_C2_)/30 μs, +*V*_C1_/50 μs, (*V*_C1_ + *V*_C2_)/50 μs, (*V*_C1_ + *V*_C2_)/20 μs, (*V*_C1_ + *V*_C2_)/20 μs.

**Figure 18:**
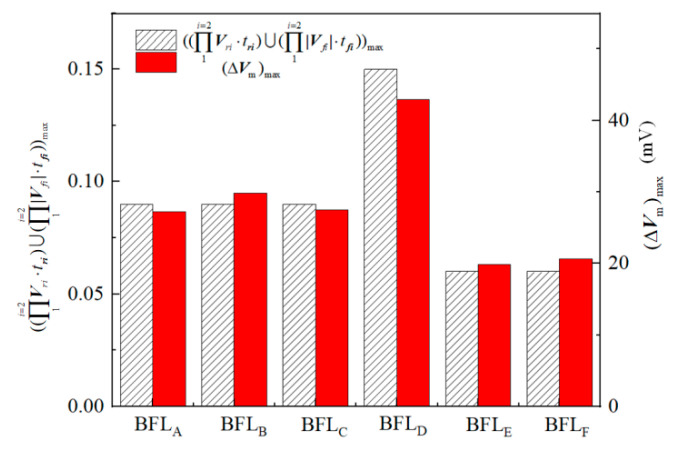
Comparison between the maximum product of discharge voltage level and its duration and the peak value of neuron membrane potential of BFEA~BFEF waveform.

The maximum value of the product of each discharge voltage level and its duration, and the peak value of neuron membrane potential Δ*V*_m_ are shown in Figure 18. It can be seen from Figure 18 that the greater the maximum value of the product of the discharge voltage level corresponding to the BFL waveform and its duration, the greater the change in neuron membrane potential under the stimulation of the corresponding waveform; The maximum peak value of Δ*V*_m_ is generated by the BFL_D_ waveform, and the maximum discharge voltage level (*V*_C1_ + *V*_C2_) lasts for 50 μs.

This conclusion can be explained by the microstructure of neurons: the accumulation of induced charges on the membrane of neurons forms the membrane potential, and the accumulation of induced charges is positively correlated with the integral of current flowing through the membrane of neurons. When the membrane current of neurons remains unchanged for a certain period, the accumulation of induced charges should be positively correlated with the product of the magnitude and duration of the membrane current of neurons [10]. Regardless of the initial charge accumulation of neurons, when the pulsed E-field stimulates neurons, the effect of an intracranial induced pulsed E-field is equivalent to the external injected current. The amplitude of the induced E-field reflects the current flowing through the cell membrane of neurons, and the discharge voltage level on the stimulation coil determines the amplitude. The duration of neuron membrane current is the duration of the discharge voltage. Therefore, the peak value of membrane potential change is positively correlated with the maximum value of the product of the discharge voltage level and its duration.

(2) Neuronal Polarizability Ratio *ξ_P_*

The neuronal polarizability under the stimulation of the BFL waveform is approximately equal to the ratio of the product of the discharge voltage corresponding to the first rising edge of the pulsed current and its holding time and the product of the discharge voltage corresponding to the second rising edge of pulsed current and its holding time:

(10) $$\varepsilon_{p}\approx \frac{V_{r1}\times t_{r1}}{V_{r2}\times t_{r2}}$$

The reason for approximate equivalence but not exact equivalence is that leakage resistances exist in the neuron equivalent model and EPMS circuit. In the first 1/4 resonant period, the pulsed current increases approximately linearly, and the induced E-field is approximately rectangular. In the actual discharge process, with the increase of the discharge time, the voltage at both ends of the energy storage capacitor will decrease slightly, resulting in the deviation Δ between the final polarizability and *V_r_*_1_·*t_r_*_1_/*V_r_*_2_·*t_r_*_2_.

It can be seen from Figure 12, Figure 13, Figure 14, Figure 15, Figure 16 and Figure 17 that the waveform of neuron membrane potential is similar to that of pulsed stimulation current. At the end of the three segments of the pulse current, the positive and negative peaks of neuron membrane potential have been reached, and the fourth segment of the pulse current will not affect the peak value of neuron membrane potential and polarizability. When *V_f_*_1_, *V_r_*_1_, and *V_r_*_2_ remain unchanged, the longer the pulse width of the stimulation current (*t_f_*_1_ + *t_r_*_1_ + *t_r_*_2_), the more the voltage drop on the energy storage capacitor, which will lead to a bigger deviation Δ. For example, the corresponding *V_f_*_1_, *V_r_*_1_, and *V_r_*_2_ of BFL_B_ and BFL_D_ waveforms are the same, but the pulse width (*t_f_*_1_ + *t_r_*_1_ + *t_r_*_2_) of BFL_B_ is 20μs longer than that of BFL_D_. Therefore, the deviation generated by BFL_D_ is higher than that of BFL_B_. The deviations of *V_r_*_1_·*t_r_*_1_/*V_r_*_2_·*t_r_*_2_ and neuronal polarizability ratio *ξ_P_* in the BFL_A_~BFL_F_ waveform are shown in Table 2.

#### 3.2.3. Comparison of BFL Waveform and Traditional TMS Waveform

There is a blind area in the deep biological mechanism of pulsed E-fields acting on neurons, and the specific requirements for positive and negative peaks of neuron membrane potential are not clear [37,38]. However, the effect of neuronal polarizability ratio on stimulation has been preliminarily concluded: neuronal polarizability ratio under the stimulation of monophasic and biphasic cosine induced pulsed E-field is about 0.84 and 0.21, respectively [12,39]. Several studies have shown that the monophasic cosine induced pulsed E-field generated a lower neuronal polarizability ratio, which brings stronger neural regulation and stimulation selectivity [18,20,21,22,23,40]. However, the efficiency of the commonly used monophasic pulsed electric discharge circuit is low because of the large energy loss, which limits the high-frequency output during repetitive stimulation [12,28]. The energy consumed by each pulse is defined as the energy difference stored in capacitor *C*_1_ and capacitor *C*_2_ at the beginning and end of the pulse:

(11) $$\Delta W=\frac{1}{2}(C_{1}{V_{C1\_\mathrm{sta}}}^{2}+C_{2}{V_{C2\_\mathrm{sta}}}^{2})-\frac{1}{2}(C_{1}{V_{C1\_\mathrm{end}}}^{2}+C_{2}{V_{C2\_\mathrm{end}}}^{2})$$

where *V*_C1_sta_ and *V*_C2_sta_ represent the discharge voltage on *C*_1_ and *C*_2_ before the pulse; *V*_C1_end_ and *V*_C2_end_ stand for the voltage on *C*_1_ and *C*_2_ after one pulse is generated.

**Figure 19:**
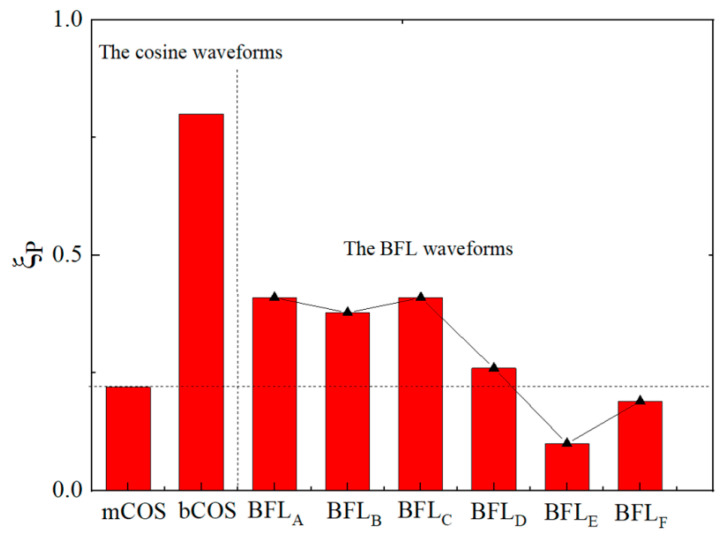
The neuronal polarizability under the stimulation of BFL_A_~BFL_F_ waveform and traditional monophasic and biphasic cosine E-field waveform.

In the BFL_A_-BFL_F_ waveform, the minimum neuronal polarizability ratio of neurons is generated by the BFL_E_ waveform. The energy loss of producing one BFL_E_ pulse is about Δ*W* = 9 J. Under the same energy loss, the monophasic and biphasic-induced pulsed E-fields generated by the EPMS circuit are recorded as mCOS and bCOS, respectively. The neuronal polarizability ratio of neurons under the stimulation of mCOS and bCOS are 0.22 and 0.80, respectively. The neuronal polarizability ratio of neurons under different BFL waveforms was compared with that of mCOS and bCOS waveforms, as shown in Figure 19.

It can be seen from Figure 16 that the BFL waveform generated by the EPMS circuit can obtain smaller polarizability (BFL_E_, BFL_F_) than the monophasic cosine intracranial induced pulsed E-field waveform. Compared with the monophasic and biphasic cosine induced E-field waveform under the same energy loss, the BFL_E_ waveform can reduce the neuronal polarizability by 54.5% and 87.5%, which means under reasonable parameter configuration, the BFL waveform can effectively reduce the neuronal polarizability and improve stimulation selectivity. In addition, since the BFL_E_ is a biphasic waveform, the energy loss after each pulse is low, and it avoids the limitation of high-frequency output during repetitive stimulation.

### 3.3. Experimental Testing

At the current stage, our team is continuing research on TMS. The implementation of the EPMS circuit and its charging circuit is still ongoing. To verify the effectiveness of this circuit design as much as possible under limited conditions, we have built a low-parameter experimental test system using the existing components in the laboratory to reproduce the stimulation waveform.

**Figure 20:**
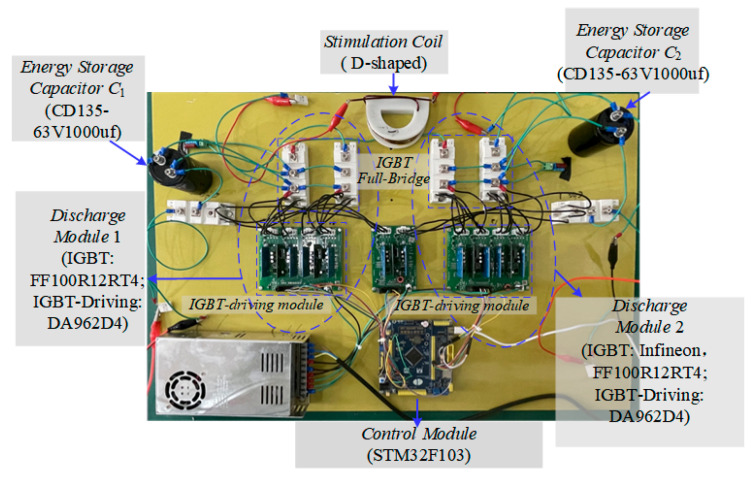
Test experimental system of the EPMS circuit (*N* = 2).

**Figure 21:**
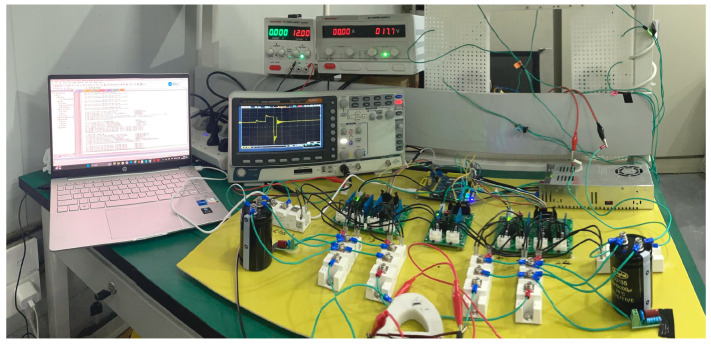
The on-site physical photo of the experimental system during the test.

Here, we used two DC power supplies to charge the energy storage capacitors C_1_ and C_2_. Figure 20 shows the low-parameter experimental test system of the EPMS circuit (*N* = 2). The models of the key components are marked in the figure. Figure 21 is the on-site physical photo of the experimental system during the test.

**Figure 22:**
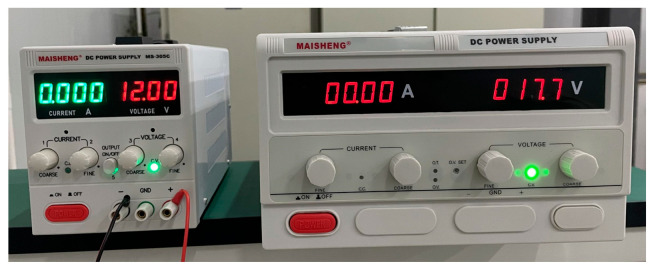
The charging voltages for C_1_ (**left**) and C_2_ (**right**).

**Figure 23:**
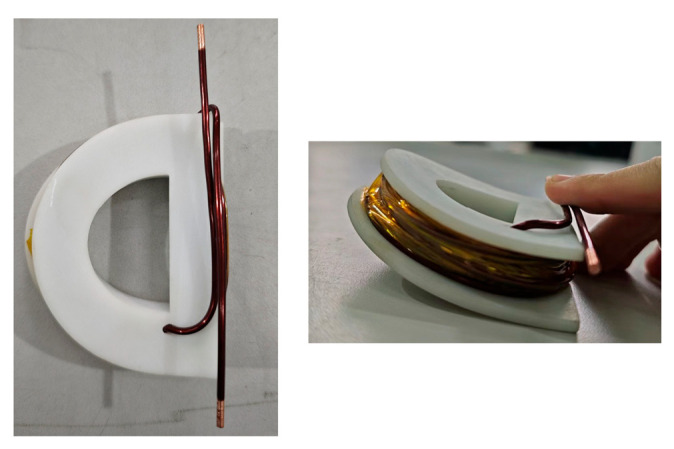
Physical photo of the D-shaped coil.

**Figure 24:**
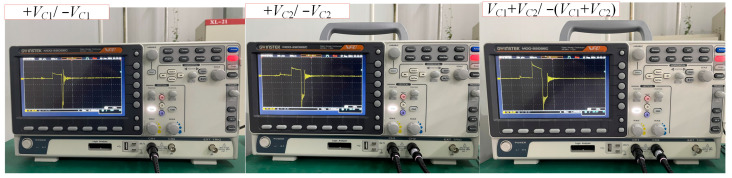
The monophasic induced E-field with adjustable amplitude.

When the charging voltages for C_1_ and C_2_ are 12 V and 17.7 V, respectively, the maximum discharge current is about 10A. Figure 22 shows the charging voltages for C_1_ and C_2_. Figure 23 is the physical photo of the stimulation coil we adopted in the test system. Figure 24 shows the stimulation waveform at +*V*_C1_/−*V*_C1_,+*V*_C2_/−*V*_C2_, and +(*V*_C1_ + *V*_C2_)/−+(*V*_C1_ + *V*_C2_) and proves that the design of the EPMS circuit can generate monophasic induced E-field with adjustable amplitude. The experiment results are in good agreement with the simulation results and prove the correctness of the circuit design.

In future studies, when the prototype experimental platform and test system commissioning are completed, we will conduct a series of safety tests and then use the research platform of the School of Life Sciences at the University of Science and Technology of China to conduct biological experiments with small rodents or humans.

## 4. Conclusions

The transient analysis of the pulsed E-field generator circuit shows that the induced pulsed E-field intensity E is proportional to the change rate d*i*/dt of the stimulation current in the pulse discharge circuit. During the first quarter resonance period of the pulse discharge circuit, the stimulation current increases approximately linearly, while the intracranial induced pulsed E-field intensity *E* nearly remains unchanged, and the induced E-field level is determined by the discharge voltage level of the energy storage capacitor. The pulse width of the induced E-field is determined by the turn-on time of the discharge switch. This conclusion shows that we can convert the physical quantity (the intracranial induced E-field) that needs to be measured after magnetic stimulation into easily analyzable electrical signals (the discharge voltage at both ends of the stimulation coil in the TMS circuit).

After the transient analysis, the EPMS circuit is proposed based on asymmetric cascade multilevel technology. The discharge voltage level is changed by cascading energy storage capacitors to form an intracranial multilevel induced E-field. Increasing the number of modules can effectively increase the number of intracranial induced E-field levels. The EPMS circuit can not only realize monophasic and biphasic cosine-shaped intracranial induced pulsed E-fields but can also realize monophasic approximate rectangular, biphasic approximate rectangular, and ladder-shaped induced E-fields.

This circuit breaks through the limitation of the traditional pulsed E-field generating circuit. Among the new induced E-field waveform produced by the EPMS circuit, the BFL waveform is taken as an example for further analysis. Combined with the neuron equivalent model, the dynamic response of neurons under the stimulation of the BFL was obtained. Results showed that the peak change in neuron membrane potential under the stimulation of the BFL waveform is positively correlated with the maximum product of the voltage level and its duration. The polarizability of neurons ξ_P_ is approximately equal to the ratio of the product of the discharge voltage level corresponding to the middle two sections of the induced E-field and its duration: *V_r_*_1_·*t_r_*_1_/(*V_r_*_2_·*t_r_*_2_).

These two conclusions have important guiding significance for the optimization of biomedical used pulsed E-field generators. It means that the biological physical quantities (neuronal membrane potential) that are difficult to measure can be transformed into easily analyzable electrical signals (the discharge voltage level and duration). Compared with the monophasic and biphasic cosine induced E-field waveform, the polarizability of neurons under the stimulation of the BFL waveform decreased by 54.5% and 87.5%, respectively. This means that the BFL_F_ waveform could effectively enhance neural regulation and improve stimulation selectivity. In addition, since the BFL_E_ is a biphasic waveform, the energy loss after each pulse is low, and it avoids the limitation of high-frequency output during repetitive stimulation.

The future research direction of our team will be devoted to the development of the experimental prototype of the multi-wave transcranial magnetic stimulator, aiming to improve the performance of the transcranial magnetic stimulation, to create conditions for further revealing the causes of mental disorders, strengthening the therapeutic effect of mental diseases, and providing the theoretical basis and technical support for the future research and development of high-efficiency intelligent transcranial magnetic stimulation equipment.

## Figures and Tables

**Table 1:** Discharge voltage level and maintenance time of different BFL waveforms.

	*V_f_*_1_ */t_f_*_1_	*V_r_*_1_ */t_r_*_1_	*V_r_*_2_ */t_r_*_2_	*V_f_*_2_ */t_f_*_2_
BFL_A_	−*V*_C2_/30 μs	+*V*_C2_/30 μs	+*V*_C1_/50 μs	−*V*_C1_/50 μs
BFL_B_	−*V*_C2_/30 μs	+*V*_C1_/20 μs	(*V*_C1_ + *V*_C2_)/30 μs	−*V*_C1_/50 μs
BFL_C_	−*V*_C2_/30 μs	+*V*_C1_/20 μs	+*V*_C1_/50 μs	(*V*_C1_ + *V*_C2_)/30 μs
BFL_D_	−*V*_C2_/30 μs	+*V*_C2_/30 μs	(*V*_C1_ + *V*_C2_)/50 μs	(−*V*_C1_ − *V*_C2_)/50 μs
BFL_E_	−(*V*_C1_ − *V*_C2_)/10 μs	+(*V*_C1_ − *V*_C2_)/10 μs	(*V*_C1_ + *V*_C2_)/20 μs	−(*V*_C1_ + *V*_C2_)/20 μs
BFL_F_	−(*V*_C1_ − *V*_C2_)/20 μs	+(*V*_C1_ − *V*_C2_)/20 μs	(*V*_C1_ + *V*_C2_)/20 μs	−(*V*_C1_ + *V*_C2_)/20 μs

**Table 2:** Comparison between *V_r_*_1_·*t_r_*_1_/*V_r_*_2_·*t_r_*_2_ and *ξ*_P_ of BFL_A_~BFL_F_.

	BFL_A_	BFL_B_	BFL_C_	BFL_D_	BFL_E_	BFL_F_
$\frac{V_{r1}\times t_{r1}}{V_{r2}\times t_{r2}}$	0.400	0.400	0.400	0.240	0.100	0.200
*ξ*_P_	0.415	0.378	0.410	0.263	0.104	0.189
Δ	3.6%	5.8%	2.4%	8.7%	3.8%	5.8%

## Data Availability

The data that support the findings of this study are available from the corresponding author upon reasonable request.
